# Systematic Spatial Bias in DNA Microarray Hybridization Is Caused by Probe Spot Position-Dependent Variability in Lateral Diffusion

**DOI:** 10.1371/journal.pone.0023727

**Published:** 2011-08-17

**Authors:** Doris Steger, David Berry, Susanne Haider, Matthias Horn, Michael Wagner, Roman Stocker, Alexander Loy

**Affiliations:** 1 Department of Microbial Ecology, Vienna Ecology Center, Faculty of Life Sciences, University of Vienna, Wien, Austria; 2 Ralph M. Parsons Laboratory, Department of Civil and Environmental Engineering, Massachusetts Institute of Technology, Cambridge, Massachusetts, United States of America; University of North Carolina at Charlotte, United States of America

## Abstract

**Background:**

The hybridization of nucleic acid targets with surface-immobilized probes is a widely used assay for the parallel detection of multiple targets in medical and biological research. Despite its widespread application, DNA microarray technology still suffers from several biases and lack of reproducibility, stemming in part from an incomplete understanding of the processes governing surface hybridization. In particular, non-random spatial variations within individual microarray hybridizations are often observed, but the mechanisms underpinning this positional bias remain incompletely explained.

**Methodology/Principal Findings:**

This study identifies and rationalizes a systematic spatial bias in the intensity of surface hybridization, characterized by markedly increased signal intensity of spots located at the boundaries of the spotted areas of the microarray slide. Combining observations from a simplified single-probe block array format with predictions from a mathematical model, the mechanism responsible for this bias is found to be a position-dependent variation in lateral diffusion of target molecules. Numerical simulations reveal a strong influence of microarray well geometry on the spatial bias.

**Conclusions:**

Reciprocal adjustment of the size of the microarray hybridization chamber to the area of surface-bound probes is a simple and effective measure to minimize or eliminate the diffusion-based bias, resulting in increased uniformity and accuracy of quantitative DNA microarray hybridization.

## Introduction

DNA microarrays, developed in the mid 1990s [1], are a widely used molecular tool for diverse biological applications such as gene expression profiling [2], [3], [4], clinical and environmental diagnostics [5], [6], genotyping [7], [8], and microbial community analyses [9], [10], [11]. Consisting of either oligonucleotide or cDNA probes immobilized on a solid surface, DNA microarrays offer a high-throughput format for the identification and quantification of thousands of different nucleic acid targets in parallel. Because microarrays are based on solution-solid-phase hybridization, their underlying hybridization dynamics are more complex than for solution-solution hybridization [12], [13]. This complexity has led to issues of data robustness and reproducibility across platforms [14], [15]. In addition to the potential for steric hindrance due to surface immobilization of probes [16], both thermodynamic and kinetic mechanisms can drive microarray hybridization dynamics in difficult to predict ways [16], [17]. Especially during static hybridization on microarray platforms without active movement of the target molecules in the hybridization solution (e.g. by shaking or pumping), mass transfer limitations and variable hybridization reaction kinetics among the different probe-target pairs impede uniform probe-target hybridization.

Generally, at the single spot scale it is thought that after an initial period where hybridization kinetics are reaction-limited, probe depletion near the spot renders the process diffusion-limited [18], although the relative importance of diffusion and reaction rates appears to also be dependent upon a number of other factors, such as the length and base composition of probes [19], [20] and the ionic strength of the hybridization buffer [21]. Hybridization efficiency even varies within a single probe spot, leading to spot-size-dependent biases [22]. Whereas these single spot biases have been relatively well-studied [22], little is known about bias at larger scales. Spatial heterogeneity can be caused by a number of factors, including uneven hybridization/washing and scanner biases (reviewed in [23], [24]). A systematic position-dependent bias is known to sometimes occur due to reproducible print tip bias, which has led to the development of print-tip normalization methods [25], [26]. Meta-analysis indicates, however, that spatial biases not related to printing processes are widespread in published microarray data [27] and in most of these cases the mechanism behind the biases remains unresolved.

In this study we identify a widespread spatial bias in microarray hybridization experiments that depends on probe spot position, and we characterize this bias experimentally using a simplified array design on standard 1”×3” glass slides. We demonstrate the underlying mechanism through a simple physical model, which reproduces the spatial bias and captures the salient features of the experimental observations. Finally, we explore the influence of hybridization conditions on the positional bias using numerical simulations. This analysis sheds light on the importance of array design and hybridization chamber geometry and suggests design modifications to minimize the spatial bias.

## Materials and Methods

### Design and fabrication of the simplified microarray

**Figure 1:**
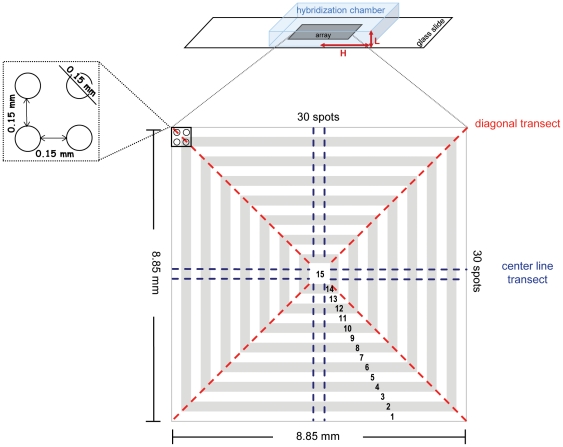
Schematic layout of the simplified, single-probe microarray. Hybridization chambers have a square footprint with all sides having a half-length *L* = 10.8 mm (21.6×21.6 mm^2^ total surface area) and a height *H* = 0.25 mm. Spot positions (1 to 15) are indicated by the alternating white and grey shading. Spots within each spot position are treated as replicate spots, with decreasing numbers of replicates (n) from spot position 1 (n = 116) to spot position 15 (n = 4). Definitions of diagonal transects (n = 4) and center-line transects (n = 8) are shown as dotted lines.

A published 16S rRNA-targeted oligonucleotide probe, DVHO831 (S-*-Dvho-0831-a-A-18, 5′-GAA CCC AAC GGC CCG ACA-3′) [28], synthesized by MWG Biotech (Ebersberg, Germany), was used for microarray spotting. The 5′ end of the oligonucleotide was tailed with 12 dTTPs (T-spacer) and the terminal dTTP was aminated to enable covalent coupling of the oligonucleotide to aldehyde group-coated glass slides (VSS-25, CEL Associates). The probe concentration was adjusted to 50 µM in 50% dimethyl sulfoxide. To exclude printing tip-related biases, microarrays were printed with a single split pin (MicroSpot 2500, Zinsser Analytic GmbH) using a BioRobotics MicroGrid spotter (Genomics Solutions) at 20°C and greater than 50% humidity. Square arrays of identical spots (approximate diameter 150 µm) were produced (30×30 spots) (Fig. 1). For testing the effect of elimination of unspotted surface area, an expanded version of the simplified array was designed such that the array covered the slide surface area for the entire hybridization chamber, which yielded a hybridized area of 5,040 spots (72×70 spots). Spotted microarrays were incubated overnight at room temperature in a wet chamber and processed with sodium borohydride, as described previously [29].

### Target preparation and labeling of the simplified microarray

The target gene was prepared and labeled fluorescently as described previously [29], [30]. Briefly, a 1.5 kB-fragment of the 16S rRNA gene of *Desulfovibrio halophilus* (DSM 5663) was amplified using the PCR primer set 616V-630R [28], [31]. The 616V primer contained a T3 promoter site tag (5′-AAT TAA CCC TCA CTA AAG GG-3′) at the 5′ end to allow T3 RNA polymerase-based *in vitro* transcription labeling of PCR products. The PCR cycling program consisted of 95°C for 3 min followed by 30 cycles of 95°C for 30 s, 52°C for 30 s and 72°C for 1 min, and concluded with 72 °C for 3 min. The target was labeled via an *in vitro* transcription reaction and subsequently template DNA was digested [29]. RNA concentration and dye incorporation rates were measured spectrophotometrically, and labeled 16S rRNA was fragmented via incubation with 10 mM ZnCl_2_ at 60°C for 30 min and was then stored in the dark at −20°C.

### Hybridization of the simplified microarray

Labeled RNA was added to hybridization buffer (6X saline sodium citrate (SSC) buffer, 10% Denhardt's reagent, 0.1% sodium dodecyl sulfate (SDS), 15% formamide) and incubated at 65°C for 5–15 min. Microarrays were sealed with the HybriWell Hybridization Sealing System (HBW2222-FL, Grace BioLabs), which have a well depth of 0.25 mm. Static microarray hybridizations, i.e. without active mixing of the hybridization solution, were performed in a custom-made hybridization chamber [28] at 42°C for 18 h, except for experiments testing the effect of hybridization time. After hybridization, microarrays were washed by shaking at RT for 5 min sequentially in a 2X SSC, 0.1% SDS buffer and a 0.1X SSC buffer, followed by 20 s in ice-cold double-distilled water. Slides were dried by centrifugation (3 min, 300 g) and stored in the dark at RT until scanning on the same day. All microarray hybridization experiments were conducted in triplicate.

### Scanning and data analysis

Slides were scanned using a GenePix Personal 4100A Array Scanner (Axon Instruments, Molecular Devices Corp.) with a 3 line average and 10 µm resolution. Photomultiplier tube gain was adjusted to just below saturation intensity of the brightest signals and identical scanning settings were used for each experimental series. Scanned images were analyzed with GenePix Pro 6.0 software (Axon Instruments) and signal intensity surface plots of microarrays were generated using ImageJ with the Interactive 3D Surface Plot plug-in [32]. Signal intensities were analyzed according to the spot position (Fig. 1) and mean relative signal intensities for each spot position were compared directly or in some cases normalized to either the maximum or minimum mean signal intensity to facilitate comparison. Poor quality spots identified by visual inspection were removed from the analysis. Statistical testing was performed with the Student's t-test. For analysis of publicly available microarrays, slide images were extracted from the Stanford Microarray Database and surface plots were created as described above. Microarrays are identifiable via their Experimental ID [33], given in the Figure legends.

### *Protochlamydia amoebophila* microarray

A *P. amoebophila* whole genome array with oligonucleotide probes was produced in the same fashion as the simplified array and hybridized using fragmented labeled genomic DNA as a target [34]. The array features several blocks with a distance of 1050 µm between blocks. Each block is composed of 144 (12×12) spots, each with a diameter of approximately 150 µm and a distance of 300 µm between spot centers. Each spot consists of 5′-amino modified oligonucleotide probes with a length of 45 to 55 nucleotides plus an additional 20 dATP spacer, which were synthesized by Microsynth (Balgach, Switzerland). Microarray fabrication was performed as described above for the simplified array, except that a higher probe concentration was used (100 µM) and arrays were printed with 16 MicroSpot 2500 split pins (Zinsser Analytic GmbH). Fragmented genomic DNA was randomly primed and labeled with Cy3 or Cy5 fluorophores by using the DecaLabel DNA Labeling Kit (Fermentas Inc.) and unincorporated nucleotides were removed using the QIAquick Nucleotide Removal Kit (Qiagen). 1–2 µg of labeled DNA was vacuum-dried (Eppendorf concentrator 5301), re-suspended in 400 µl of hybridization buffer (35% formamide, 5x SSC, 0.1% SDS, 0.1% *n*-lauryl sarcosine, 0.1% blocking reagent, 50 µg ml^−1^ salmon sperm DNA), and denatured at 95°C for 10 min. Slides were pre-hybridized with blocking reagent (5x SSC, 0.1% SDS and 1% bovine serum albumin) for at least 2 hours at 42°C, and were then washed with double-distilled water and with isopropanol and finally dried by centrifugation. Slides were hybridized using sealed coverslips with a 0.25 mm well depth (HBW2260, HybriWell Sealing System; GRACE Bio Labs) under constant shaking at 400 rpm (ThermoTWISTER Comfort, QUANTIFOIL Instruments GmbH) for 16 h at 42°C. Slides were washed at 42°C with three serial washes of increasing stringency (2x SSC, 0.1% SDS/0.1x SSC, and 0.1% SDS/0.1x SSC), rinsed with double-distilled water, and air-dried. Images were recorded by scanning the slides with a GenePix Personal 4100A array scanner (Axon Instruments, Molecular Devices Corporation). Hybridization images were used directly for creation of signal intensity surface plots with the ImageJ software.

### Microarray data

The microarray data have been deposited in the NIH National Center for Biotechnology Information's Gene Expression Omnibus database (GEO, www.ncbi.nlm.nih.gov/geo/) under accession number GSE26275.

### Modeling

It has previously been demonstrated that for target-limited conditions and hybridization times up to 12–24 h the hybridization reaction timescale is markedly shorter than the diffusion timescale [35]. Consequently, reaction terms can be neglected in describing the spatiotemporal evolution of the target concentration and a Fickian diffusion model accurately captures the dominant dynamics of the full diffusion-reaction model [35]. This simplification is supported below by a rigorous scaling analysis, which we hope might be of interest also to future hybridization studies.

Thermodynamic models, such as those based on Langmuir adsorption theory, are a popular approach to modeling microarray hybridization [36], [37], [38], [39]. Equilibrium Langmuir-based models, however, cannot adequately describe the spatial bias observed in this study, because all spots in our simplified array consist of the same probe molecules with identical thermodynamic properties (e.g. hybridization free energy) and would therefore be predicted to have identical signal intensity by thermodynamic models. Extensions of the Langmuir model that take into account the effect of probe depletion [40] are also inadequate for this purpose because even in the case of global probe depletion, in which the behavior of each spot affects other spots, an assumption of perfect mixing is levied that disallows the possibility of location-dependent phenomena.

Extensions of Langmuir-based models to diffusion-reaction models are necessary to capture spatial variations because they incorporate transport processes [35], [41]. Gadgil and co-workers proposed and solved such a model [35]. In the bulk fluid, reaction is irrelevant and target dynamics are described by the diffusion equation [35]. Close to the spot surface, both diffusion and reaction could in principle be important. For target-limited conditions and hybridization times up to 12–24 h, Gadgil et al. [35] found that the hybridization reaction timescale is markedly shorter than the diffusion timescale. Diffusion is thus negligible near the spot, where dynamics are dominated by reaction. This justifies the use of a perfect absorption boundary condition (*C* = 0) at the spot surface. To show this explicitly, we present a formal scaling analysis that demonstrates the smallness of a dimensionless parameter, *P*, measuring the ratio of the hybridization timescale to the diffusion timescale.

The diffusion-reaction equation for the concentration of target, *C*, is
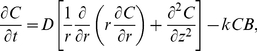
(1)where *D* is the diffusion coefficient of the target, *r* and *z* are the radial and vertical coordinates, *k* is the hybridization rate constant, and *B* is the concentration of probe. In equation (1), we have not included the dissociation of bound target strands because an assumption of irreversible hybridization kinetics is justified for perfectly complementary hybridization [6], as is the case in our experiments. It is useful to derive a dimensionless form of equation (1) by introducing dimensionless variables (denoted by a hat) as follows:
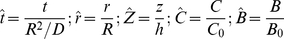
(2)


The characteristic scales used to make variables dimensionless are the spot radius *R*, the chamber height *h*, a reference probe concentration *B_0_*, an (arbitrary) reference target concentration *C_0_*, and the diffusion timescale *R^2^/D*. Equation (1) can be written in terms of the dimensionless variables as,
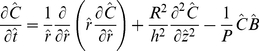
(3)where the dimensionless parameter 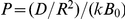 represents the ratio of the hybridization timescale 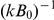 to the diffusion timescale 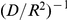. Incidentally, we note that the limiting timescale for diffusion is the one pertaining to diffusion in the radial direction, rather than in the vertical direction, since in our case (and in [6]) *R*<*h* (if instead one had *R*>*h*, one would have to redefine *P* as 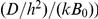.

The value of *P* determines whether it is justifiable to neglect either reaction or diffusion in the vicinity of the spot. For *P*≫1, reaction is too slow to matter and can be neglected, whereas for *P*≪1 reaction is much faster than diffusion, and the latter can be neglected. Most hybridizations fall in the latter category. Importantly, *P*≪1 in equation (3) yields the condition, 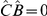, which corresponds to a boundary condition of perfect absorption at the spot surface (*C* = 0).

For example, for the case considered by Gadgil et al. [35] (*R* = 50 µm, 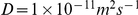, 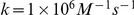., 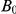, = 78 µM), we find a reaction timescale 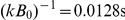 and a diffusion timescale, (*D*/,*R-2.*)-*−1*.* = 250 s*, thus obtaining *P* = 5×,10-*−5*.<*<1*. The smallness of this value supports the conclusion – obtained by Gadgil et al. [35] through full integration of equation (1) – that at the spot surface reaction dominates over diffusion and thus *C* = 0.

The same result applies to the system under consideration here. We performed a careful analysis of the parameters involved by resorting to the relevant literature (we note that our values are not the same as those of Gadgil et al. [6], though these differences have little effect on the end conclusion). The radius of our spots was *R* = 75 µm. We used a diffusion coefficient of *D* = 1×,10-*−9*., m-*2*., *s*-*−1*., based on published estimates for single-stranded RNA [42]. We estimated *k* according to the Wetmur-Davidson equation [43] to be 5.0×,10-*4*., M-*−1*., *s*-*−1*. Although *k* is known to be sensitive to several factors [44], this value agrees well with experiments [45]. To estimate *B_0_*, we followed the method of Cheung et al. [46] and assumed that the spot is originally hemispherical with radius *R* and has a concentration *B*_S_, then dries to a disk of the same radius and height *h*_D_, while conserving the total amount of probe. Assuming *h_D_* = 2 µm [35] and using the value *B_S_,* = 50 µM from our experiments, this yields, *B_0_* = (2/3)(*R*/,*h_D_.*),*B_S_* = 1.25 mM. Using these values, we find a reaction timescale 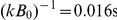. and a diffusion timescale 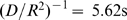, thus obtaining *P* = 2.8×,10-*−3*.≪*1*. It is thus justified to assume that the spot acts as a perfect sink, where *C* = 0.

We solved the diffusion equation describing the target concentration under conditions representing the essential features of the chamber's geometry and operation, with the goals of (i) understanding the dominant process responsible for hybridization heterogeneity, and (ii) exploring the role of geometrical parameters in determining the magnitude of this heterogeneity. For this purpose, we solved the three-dimensional unsteady diffusion equation
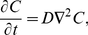
(4)where *C* is the concentration of the target, *D* is the target's diffusion coefficient, *t* is time, and ∇^2^ is the Laplacian operator.

Equation (4) was solved numerically using a finite-element code (COMSOL Multiphysics) for the dimensions of the simplified microarray (Fig. 1), with the exception that the chamber height assumed in the numerical simulation was 150 µm or 750 µm. In detail, the numerical model represents a hybridization chamber of half-width *L* and height *H*, containing a square block of 30×30 spots that covers an area of *M*×*M* mm^2^. Each spot had a diameter of 150 µm and the inter-spot distance is 150 µm. Hence, the 30×30 spots covered 8.5×8.5 mm (*M* = 8.5 mm). Both the height of the chamber and the relation between *M* and *L* will be seen to be important in determining hybridization heterogeneity.

Each simulation was carried out over 24 hours, with a diffusion coefficient of 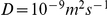. Importantly, a rescaling of the diffusion coefficient is simply equivalent to a rescaling of time, because the diffusion equation is linear. For example, if the diffusion coefficient is halved (larger target molecules), results will apply unchanged, except that all times will be doubled. The simulation proceeds by solving the three-dimensional diffusion equation in the entire chamber at subsequent instants in time. The initial condition was *C* = 1 in the entire chamber, i.e. a constant concentration of target (well-mixed). Because the diffusion equation is linear in the concentration, the solution is independent of the particular initial target concentration (allowing us to set *C* = 1 without loss of generality), unless the concentration is so high that individual spots become saturated (a case we do not consider here).

For each spot, the flux of target strands to the spot was calculated at each instant in time and subsequently integrated over the total time (24 h). For this, the boundary condition imposed at the spot was one of perfect absorption (*C* = 0), consistent with reaction being considerably faster than diffusion, as detailed above. The flux was thus computed based on Fick's law as –*DA*(∂*C*/∂*z*), where ∂*C*/∂*z* is the gradient of target concentration normal to the spot and *A* is the surface area of the spot. This yielded the total amount of target that had been bound to the spot over the simulation time. At all other surfaces (top, bottom and side walls of the chamber), which were impermeable to the target, no-flux boundary conditions were imposed.

Three different cases were considered. The first is the closest to the simplified array described above, with a large and shallow hybridization chamber (*L* = 10.8 mm, *H* = 150 µm), differing from the real one only in the chamber height (the actual chamber has *H* = 250 µm). The second models a large and deep hybridization chamber (*L* = 10.8 mm, *H* = 150 µm), to explore the effect of chamber height. The third case is that of a small and shallow chamber (*L* = 4.5 mm, *H* = 150 µm), to understand the effect of the amount of surface area not covered with spots. In all cases, the number of spots and the area covered by the spots are the same.

## Results and Discussion

### The ‘boundary-effect’: Increased signal intensity at microarray boundaries

**Figure 2:**
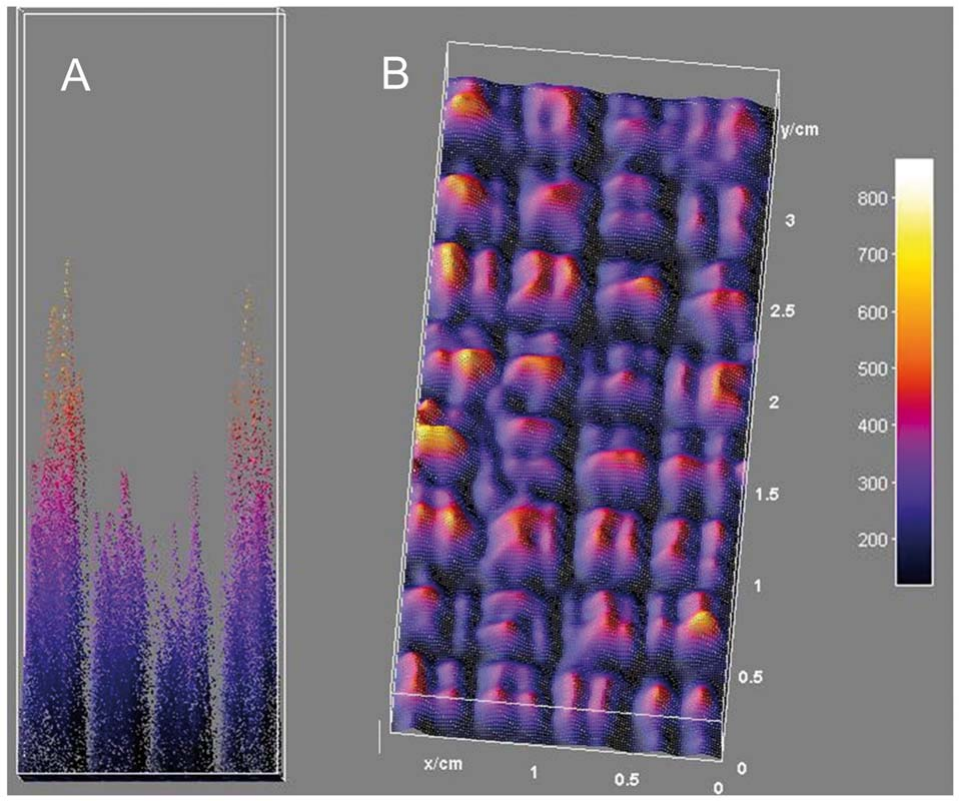
Spatial variations in fluorescence intensity of labeled genomic DNA hybridized to a genome array of *Protochlamydia amoebophila.* Signal intensities from 32 blocks are displayed, with each block composed of 144 (12×12) spots. Signal intensities (expressed in arbitrary fluorescence units) are shown as height and color heatmap in a three dimensional surface plot and displayed as (A) lateral view and (B) top view. An increased boundary signal results in a U-shaped intensity profile across the entire array (A). A tendency for increases in boundary signals is also evident at the boundary of blocks (B), though the pattern is somewhat obscured by additional signal intensity variations caused by different probes at different positions.

**Figure 3:**
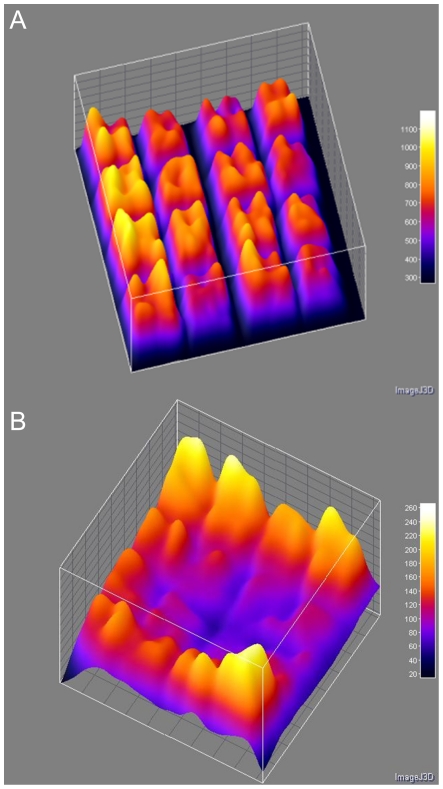
Two examples of spatial variation in signal intensities from publicly available microarray images. Signal intensities are shown as height as well as color heatmap on a three dimensional surface plot. (A) Channel 2 image from a *Vibrio cholerae* comparative genome hybridization experiment (genomic DNA targets) on a microarray consisting of 16 (4×4) blocks and 272 (17×16) spots per block (ExpID 68809) (B) Channel 1 image from a comparative gene expression hybridization experiment (cDNA targets) on a *Mycobacterium tuberculosis* microarray that consists of 16 (4×4) blocks and 289 (17×17) spots per block (ExpID 75165). Both arrays were hybridized overnight without agitation. Complete experimental details are available at the Stanford Microarray database under the associated experiment ID.

During the analysis of a whole genome microarray of *Protochlamydia amoebophila* [34], we observed a marked and reproducible spatial variation in fluorescence signal intensity, despite the fact that the microarray was agitated during hybridization to achieve a more uniform distribution of target molecules over the array surface. This variation was characterized by a systematic increase in signal intensity of spots located at the boundary of the spotted area of the slide and along the borders of individual sub-blocks (Fig. 2A, B). A random search of publicly-available microarray datasets deposited in the Stanford Microarray Database revealed the same trend in other DNA microarray hybridization experiments (Fig. 3). It has previously been reported that non-random spatial variations in target signal intensity are found in several microarray platforms [27], suggesting that the observed effect may represent a widespread and as yet unexplained feature of microarray hybridizations that is independent of target organism and platform.

**Figure 4:**
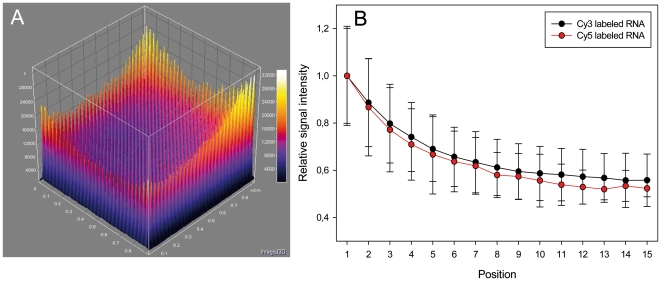
Probe spot position-dependent spatial variation in signal intensity in the simplified microarray hybridized with labeled RNA (single-color) under target limiting conditions for 18 h. (A) Mean signal intensities of three replicate hybridizations using Cy3 labeled RNA are shown as height in a three dimensional surface plot. (B) Microarray hybridization of Cy3 (red) and Cy5 (blue) labeled target RNA. Mean relative signal intensities are given as fraction of the spot position with highest absolute signal intensity and are displayed as function of the respective position. Error bars represent relative standard deviation per spot position for three replicate hybridizations.

**Figure 5:**
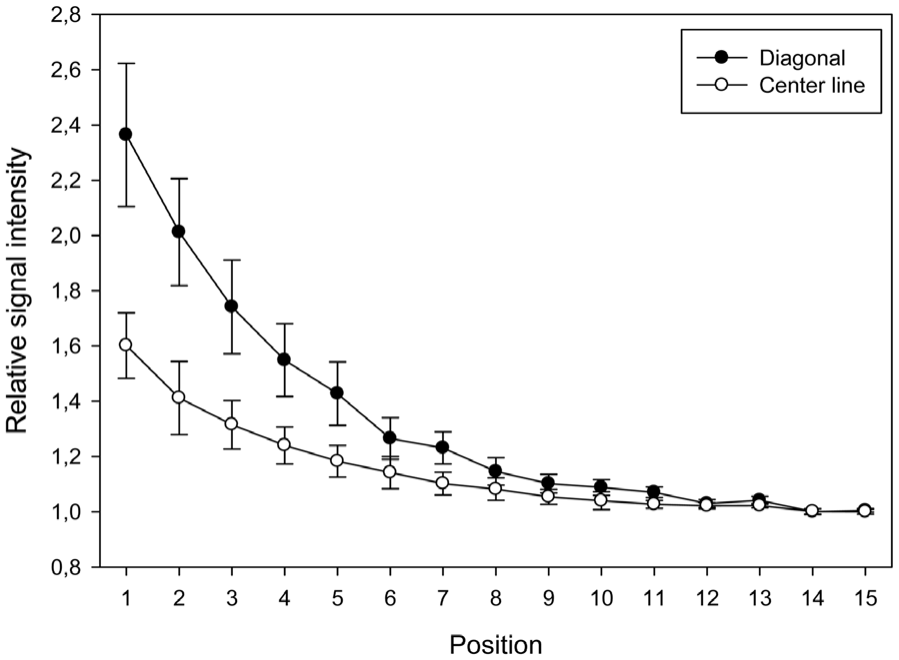
Comparison of relative signal intensities of diagonal (black) and center line (white) transects on the simplified microarray (see Fig. 1). Labeled RNA was hybridized under target limiting conditions for 18 h. Mean relative signal intensities for each position are normalized to the lowest value of all positions. Error bars represent relative standard deviations of the mean signal intensities per spot position for three replicate hybridizations.

In order to systematically investigate this phenomenon and to exclude other biases previously attributed to spatial intensity variations [25], [26], we employed a simplified square array of 900 identical spots of the same probe (Fig. 1) hybridized under target-limiting conditions typical of quantitative microarray hybridization (300 ng for 18 h). We found that this setup reproduces the spatial variation observed in more complex DNA microarrays (Fig. 4A, B). Marked spatial variations were observed with both Cy3- and Cy5-labeled target RNA (each hybridized separately with a microarray) ruling out dye-specific effects (Fig. 4B). The increase in fluorescence signal intensity was apparent in the outermost positions of the array and reached a maximum average intensity at corner positions (Fig. 5), suggesting that the driver for spatial variation in signal intensity was a limitation of the mass transfer to the center of the array block. Similarly, there are reports of increased hybridization efficiency at microarray spot edges for individual spots [22] as well as increased boundary signal intensity in polyacrylamide gel immobilized oligonucleotide hybridization cells [47], which was attributed to a process of retarded diffusion through the gel matrix [47]. While analogous to our observations, these studies represent a boundary effect at a much smaller scale (∼100 µm) than the spatial gradients we observed, which occur over the scale of the blocks of spots (generally several mm; 8.85 mm for the simplified array block). Though the simplified single probe array does reproduce the spatial variation seen in conventional multi-probe arrays (Fig. 2 and 3), it would be anticipated that the variation in number and location of spots with different, possibly competing probes in complex multi-probe arrays would also affect the magnitude and characteristics of this phenomenon.

### Lateral diffusion causes a systematic spatial bias

**Figure 6:**
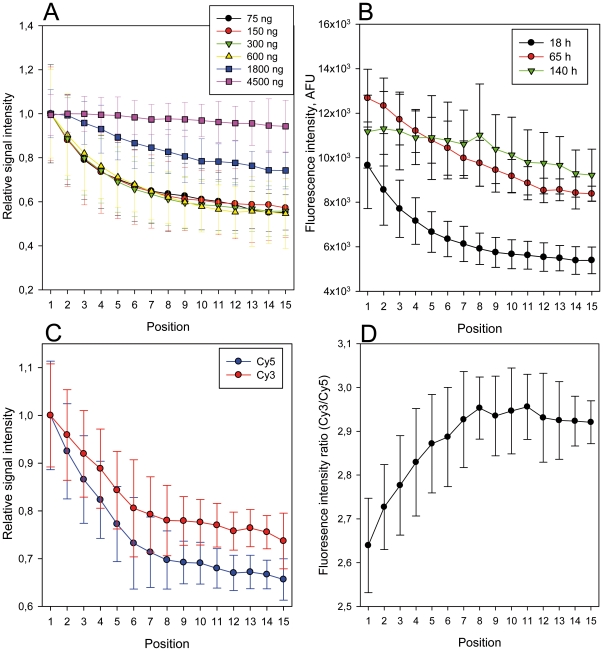
Factors affecting the boundary bias and implications for signal ratios in competitive hybridizations. (A) Hybridization of labeled RNA (single-color) at a range of concentrations (75–4,500 ng) for 18 h. (B) Hybridization of labeled RNA (single-color) under target limiting conditions for 18, 65 and 140 h. (C) Competitive two-color hybridization of Cy3- and Cy5 labeled RNA mixed at approximately 3∶1 ratio (900 ng and 300 ng, 18 h hybridization). (D) Ratios of signal intensities from competitive hybridization in C. Signal intensities for A and C are normalized to the spot position with the highest signal and error bars for A to D indicate standard deviation per spot position for three replicate hybridizations.

In order to test probe-limiting, rather than target-limiting, conditions, we hybridized the simplified array with several concentrations of target RNA (18 h hybridization). Increasing the target concentration reduced the boundary effect and could even quench it at high levels (Fig. 6A). The concentration of target affects both diffusion rates and hybridization rates, so in order to discern whether hybridization equilibrium plays a role in the observed effect we conducted additional hybridizations under target-limiting conditions for 18, 65, and 140 h (Fig. 6B). The mean signal intensity of the outer spots (position 1) increased insignificantly between both 18 and 65 h (P = 0.09) and 18 and 140 h (P = 0.36), suggesting that hybridization equilibrium had almost been reached for these spots within 18 h. In contrast, the signal intensity of the inner spots increased dramatically between 18 and 65 h (e.g. P = 0.002 for position 15), indicating that after 18 h the inner spots are much further away from hybridization equilibrium than the outer spots (Fig. 6B). The overall effect of hybridization time on the position-dependent change in signal intensity was a quenching of the boundary effect at longer hybridization times. The different behavior of inner and outer spots during extended hybridization times supports the idea that the boundary effect is driven by spatial variability in lateral diffusion present under diffusion-limited non-equilibrium hybridization conditions, typical for most common microarray applications, like gene expression analysis and microbial diagnostics [2], [3], [5], [6].

The variation of lateral mass transfer with position in the array raises the possibility that ratio signal intensities calculated from competitive two-color hybridization experiments, which for example are performed to measure relative differences in gene expression, may be sensitive to probe location when the competing target nucleic acids are present at different concentrations. To test this, we mixed identical targets labeled with either Cy3 or Cy5 at a 3∶1 ratio (900 ng/300 ng) and competitively hybridized them under standard conditions (18 h hybridization) (Fig. 6C). We observed that signal intensity ratios are indeed sensitive to location, with increased ratios for inner spots compared to outer spots (P<0.001 for comparison of positions 1 and 15) (Fig. 6D). While the targets were mixed at a ratio of 3∶1, the actual target ratio was presumably 2.6∶1 because this was the ratio measured at the boundary spots (Fig. 6D). Relative to this, spots at inner positions had elevated ratios, with a maximum of 2.9∶1 in the center of the block. These results are consistent with the concept that diffusion limitation under non-equilibrium conditions leads to under-representation of low abundance signals [48], [49], which causes over-estimates of intensity ratios and thus false relative abundance results.

### Modeling reveals the critical role of hybridization slide design and chamber geometry

Because transport within the chamber of our simplified microarray occurs only by molecular diffusion and spots do not cover the chamber surface area uniformly (there is typically a spotless area surrounding a square array of spots), we hypothesized that the boundary bias results from the outer spots in the array having a larger supply of target strands coming from the outer spotless area. We thus developed a mathematical model based on Fickian diffusion to predict hybridization behavior for the simplified array design and found that it reproduces the fundamental features of the observed behavior. Inner spots get less of their mean share of the target strands from the outer, spotless area, because they are in the shadow of outer spots. This, then, leads to the predictions that (i) the amount of spotless area is an important determinant of the bias; therefore in the limiting case of a chamber that is entirely covered by spots, we expect no bias, and (ii) deeper chambers cause less bias, because greater depths alleviate the shadowing effect and favor a more equal partitioning of the target strands from the outer, spotless area to all spots.

**Figure 7:**
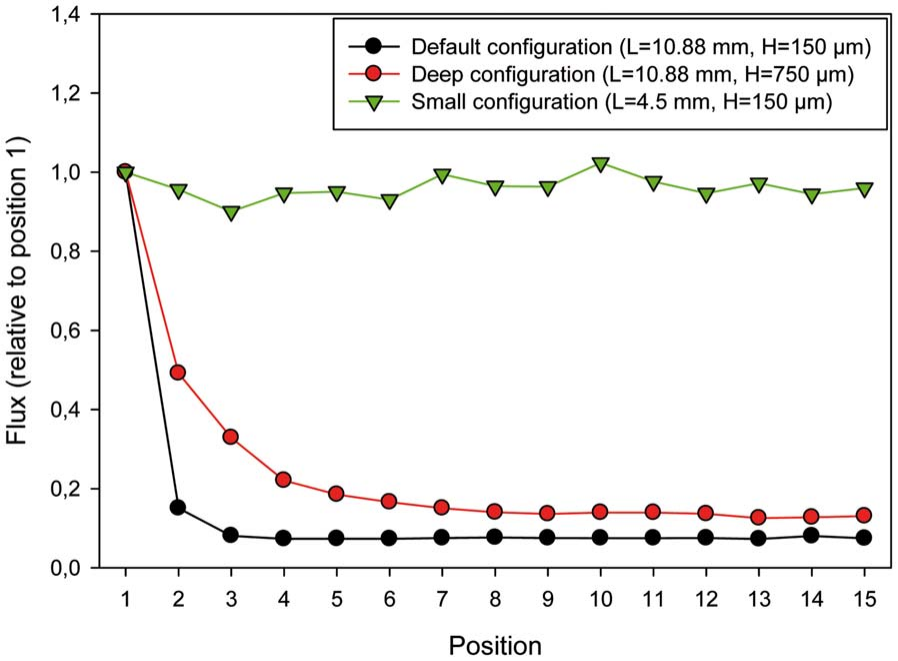
Numerical simulation of the three-dimensional unsteady diffusion equation to simulate flux of labeled RNA to each probe position over a 24 h hybridization. Three different cases were considered: a large and shallow chamber, similar in dimensions to the simplified array used in experiments (‘default configuration’, black), a large and deep chamber (‘deep configuration’, red) that tests the effect of increasing the chamber height, and a small and shallow chamber (‘small configuration’, green) that tests the effect of unspotted surface area. The number of spots and the spotted area are the same for all simulations.

**Figure 8:**
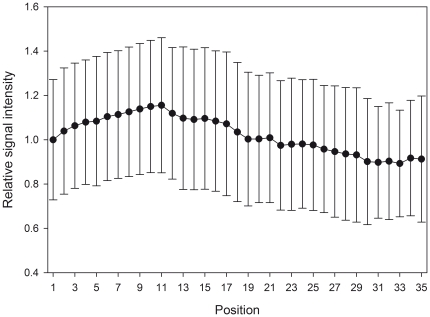
Effect of eliminating unspotted surface area on the probe spot position-dependent signal intensity. 300 ng of Cy3-labeled RNA was hybridized for 18 h. The hybridization chamber geometry was the same as previous experiments. The spot grid of the simplified 30×30 array was expanded to reach the edges of the hybridization chamber, which yielded an array of 5,040 spots (72×70). Spot position was calculated identically as for the 30×30 array, with the outer position being one and the inner position equaling 35. Signal intensities are normalized to spot position one and error bars indicate standard deviation per spot position for five replicate hybridizations.

To test these predictions concerning the design of the hybridization slide and chamber geometry, we compared mathematical modeling results for three configurations (Fig. 7): a large and shallow chamber (‘default configuration’), a large and deep chamber (‘deep configuration’), and a small and shallow chamber (‘small configuration’) (see Materials and Methods). This analysis revealed that chamber geometry plays an important role in determining the magnitude of the boundary effect. For the default configuration, the closest to the actual chamber, the bias is strong, with innermost spots subject to a reduced flux of as little as 7% of the outermost spots over 24 hours (Fig. 7, black line). The deep configuration reduces the bias somewhat (Fig. 7, red line); more of the target strands from the outer, spotless region reach the inner spots without being intercepted by the outer spots, yet flux to innermost spots is still only 12% of the outermost ones. Hence, chamber shallowness is one geometric factor that favors boundary bias. However, by far the strongest control on the bias is exerted by the amount of spotless area (the area of the chamber that lies outside the array of spots). Indeed, in the small configuration (Fig. 7, green line), where the amount of spotless area is zero, the boundary bias vanishes and the flux to each spot is nearly the same (within numerical error). In order to empirically evaluate the modeling prediction, we generated an expanded version of the simplified array featuring an array of 72×70 spots that covered the entire surface area of the hybridization chamber, thereby eliminating unspotted area between the boundary of the array and the hybridization chamber wall. As predicted by our diffusion model, removal of unspotted area by extending the array of spots to the hybridization chamber wall was sufficient to eliminate the spot position-dependent bias in signal intensity when hybridized under identical conditions to the 30×30 array (Fig. 8). This conclusively proves the role of the spotless area in generating and controlling the magnitude of the boundary bias. In summary, the boundary effect can be mitigated by choosing a deeper chamber and – most importantly – covering the entire bottom surface area of the chamber with spots.

Another option for minimizing the boundary effect is to increase hybridization time to equilibrium, though this may be prohibitively long in cases where the target concentration is very low. Alternatively, hybridization systems with active mixing generally enhance target delivery and hold the potential to reduce diffusion limitations and thereby minimize the boundary effect. However, the mechanism by which active mixing is ensured differs greatly among the different hybridization systems (such as pressure-driven flow-through or surface acoustic wave micro-agitation systems) [50], [51], [52] and not all systems achieve homogenous mixing of the hybridization solution [53]. The rate and path of the hybridization fluid flow over the microarray surface ultimately determines whether all spots experience the same influx of complementary target molecules. Heterogeneous active mixing can thus also cause a spatial bias in surface hybridization. An optimal active mixing device for microarray hybridization would thus need to ensure sufficient uniformity in the convection of target molecules [53]. Data normalization, for example based on technical probe spot replicates that are selectively positioned on the microarray surface [54], is another potential possibility to account for spatial biases in probe signal intensity.

### Conclusions

We observed and explained systematic spatial gradients in nucleic acid surface hybridizations, both experimentally and theoretically, and found that heterogeneity in lateral diffusive fluxes generate a consistent position-dependent bias in target signal intensity in single-color hybridizations as well as intensity ratios in competitive two-color hybridizations. A simple diffusion model supported the conclusions drawn from the experimental data and revealed that the geometry of the hybridization chamber and the probe spot area play an important role in determining the intensity of the spatial bias. The benefits realized from optimal adaptation of probe spot area to hybridization chamber size are a shift from diffusion-limited to reaction-limited conditions and increased microarray hybridization uniformity. Many commercially available hybridization systems with active mixing (another potential solution to diffusion limitation) are expensive and lack proof of a homogenous distribution of target molecules, which is required to effectively reducing diffusion limitation problems during hybridization. Our proposed strategy, adjusting the geometry of the hybridization chamber to the surface area of spotted probes or vice versa, is a relatively simple and cost-effective solution to diminish biases resulting from diffusion limitation and enhance accuracy of microarray-based quantification even without active mixing.
